# The role of computerized tomography angiography in the management of cases undergoing penile revascularization due to erectile dysfunction: prospective cohort study

**DOI:** 10.1186/s12880-022-00941-z

**Published:** 2022-12-08

**Authors:** Fatih Akdemίr, Önder Kayıgίl, Oktay Algın, Ali İpek

**Affiliations:** 1 Department of Urology, Terme State Hospital, Terme, Samsun Turkey; 2 Department of Urology, Faculty of Medicine, Yıldırım Beyazıt University, Bilkent, Ankara, Turkey; 3 Department of Radiology, Faculty of Medicine, Yıldırım Beyazıt University, Bilkent, Ankara, Turkey; 4 Department of Radiology, Bilkent City Hospital, Bilkent, Ankara, Turkey; 5 Ünye, Turkey

**Keywords:** Erectile dysfunction, Surgical treatment, Penile revascularization, Computed tomography angiography

## Abstract

**Background:**

In this study, the role and efficiency of computerized tomography angiography (CTA) in the postoperative management of patients with penile revascularization were evaluated.

**Methods:**

Between 2014 and 2018, penile revascularization surgery was performed in 78 patients who presented with the complaint of erectile dysfunction (ED). The mean age of the patients was 47.17 ± 13.26 (23–69) years. Patients with a regular sexual partner and relationship, who hadn’t benefitted from medical treatment and who had ED complaints for at least three months were included in the study. The cases were divided into three groups according to their age (20–40, 41–60, and > 61 years). All the cases were evaluated preoperatively using the five and 15-item International Index of Erectile Dsysfunction (IIEF-5 and IIEF-15) questionnaire, cavernosometry, corpus cavernosum electromyography, and penil color doppler ultrasonography. At the postoperative third month, IIEF 5–15 questionnaire was repeated and anastomotic patency was evaluated by performing CTA scanning.

**Results:**

CTA performed at the postoperative third month revealed anastomosis patency in 56. In 22 cases, the anastomosis area could not be observed. Among the patients with anastomosis patency, the rate of the IIEF-5 increase in the postoperative period was between 35.0 and 80.8%, while in those patients without anostomotic patency, the increase rate of IIEF-5 were between 12.5 and 23.3%. Increases in the IIEF-5 and IIEF-15 questionnaire scores were found to be significantly higher in the group in which anastomotic patency was observed on CTA compared to remaining patients.

**Conclusion:**

The CTA results and changes in the IIEF rates after penile revascularization had a high correlation. Anastomotic patency with CTA can guide the timing of more invasive procedures such as penile prosthesis implantation.

## Background

Erectile dysfunction (ED) can be defined as the inability to achieve and/or maintain erection for satisfactory sexual performance [1]. There is an increase in the global prevalence of ED. According to the Massachusetts Male Aging Study data, ED is seen at varying degrees in more than 52% of men aged 40–70 years [2]. It has been reported that in more than 70% of cases without endocrine or neurological disorders, ED has an organic origin and is caused by hemodynamic factors and arterial or venous insufficiency. In cases where an organic etiology is suspected, the penile anatomy, physiology, and hemodynamics should be well evaluated. ED is assessed using methods such as penile color Doppler ultrasonography (PCDU), dynamic pharmacocavernosometry, selective pudendal pharmacovigilance, nocturnal penile tumescence test, and corpus cavernosum electromyography (CC-EMG) [3, 4]. Computed tomography angiography (CTA) is a rapid, non-invasive imaging technique used to diagnose vascular diseases, show the relationship between non-vascular diseases and vascular structures, and reveal the vascular anatomy; thus, this method can be useful in identifying patients who require endovascular treatment. Various publications report that CTA is an effective and reliable method for the diagnosis and treatment of priapism, arterial injury, and ED, as well as detecting venous leakage in venogenic ED [5–8]. In this study, CTA scan was performed using intra-cavernous contrast material after penile revascularization surgery. This allowed for the investigation of not only the correlation between CTA findings and erectile function (EF) but also the usability of CTA as a non-invasive method in evaluating the penile anatomy and vascular structures in patients that had undergone penile revascularization surgery.

## Methods

Our study was designed as a prospective cohort study. The study protocol was approved by the local ethics committee, and all the patients signed an informed-consent agreement form. Patients also gave consent for the publication of surgery photographs and CTA images. In this study, penile revascularization surgery was performed on 78 patients with ED of organic origin between June 2014 and September 2018. The mean age of the patients was 47.17 ± 13.26 (23–69) years. The patients were divided into three age groups: Group 1 (20–40 years), Group 2 (41–60 years), and Group 3 (> 60 years). According to their anamnesis, there were diabetes in 15 cases, smoking in 18 cases, hyperlipidemia in 11 cases and cardiovascular disease in 9 cases. In addition, the body mass index of nine cases was above 26. Inclusion criteria for the study were having had a regular sexual partner, having ED for at least three months, not benefiting from medical treatment, and the presence of non-psychogenic ED. In order to exclude ED of psychogenic origin, the patients were referred to an experienced psychiatrist, and those with stress, general anxiety, depression, obsessive–compulsive disorder, performance anxiety, relationship problems, feelings of guilt, loss of self-confidence, sexual phobias, or any other psychopathology were excluded from the study. After obtaining the detailed anamnesis and genital examination of the patients, their complete blood count, fasting blood glucose, lipid profile, luteinizing hormone, testosterone and prolactin levels were determined and the blood pressure values were measured. PCDU, CC-EMG and cavernosometry were preoperatively performed in all the patients for diagnostic purposes. In preoperative PCDU cases with a peak systolic velocity (PSV) value of less than 25 cm/s and/or end diastolic velocity (EDV) value of higher than 5 cm/s were evaluated based on both the cavernosometry and CC-EMG results, and then the distinction of arterial and/or venous insufficiency was made. In addition, the diameters and flow velocities of the inferior epigastric artery (IEA) were evaluated to determine the appropriate side for anastomosis. All the cases were also assessed using the five and 15 item version of the International Index of Erectile Dysfunction (IIEF-5, and IIEF-15) qusetionnaire and the IIEF-EF domain preoperatively.

PCDU (BK Medical, Herlev, Denmark) was conducted using a linear transducer probe (8 MHz) to evaluate penile vascular abnormalities. First, papaverine hydrochloride (60 mg) was injected into one of the cavernous bodies of the penis. Then, the patient was allowed to rest in a comfortable room for 20 min. Arterial insufficiency was defined as a systolic blood flow of less than 25 cm/s, in PCDU. End-diastolic arterial blood flow greater than 5 cm/s was defined as veno-occlusive dysfunction.

To evaluate CC-EMGs, penile cavernous electrical activity (CEA) was recorded using a high-speed electromyography module equipped with a computer (Medical Measurement Systems, Enschede, the Netherlands). The sampling frequency was 200 Hz, and a band-pass filter with a cut-off frequency of 0.1–20 Hz was used. During the CC-EMG recordings, monopolar needle electrode was used to measure the cavernous electrical activity. A grounding electrode was placed to the patient's foot to avoid electrical activity simultaneously originating from non-penile areas. It appears as a single line in the EMG recording. CC-EMG recordings were started after the patients rested for 10 min in a quiet and dim room. CEA potentials were recorded for 10 min. Later the CEA potentials of the penile cavernous nerves was assessed by detecting the peak-to-peak amplitudes. Ten minutes later, papaverine hydrochloride (60 mg) was injected into a single cavernous body for avoiding the pattern of discoordination, which manifested by an increase or no difference in the CEA recording following the injection and suggested the neurogenic ED.

After CC-EMG recordings were made, cavernosometry was conducted with the same device. In the presence of the following criteria, a diagnosis of caverno-occlusive dysfunction was made.

1. Requires a maintenance flow rate greater than 5 ml/min after revealed an intracavernous pressure of 150 mmHg with the artificial erection test.
2. The intracavernous pressure decreased by a minimum of 45 mmHg within 30 s following the termination of infusion.

Before the operation, all the patients signed an written informed consent form including detailed information about the revascularization operation. The operations, were conducted using the Furlow-Fisher procedur, of the Virag–V technique [9, 10]. Unlike the Furlow–Fisher procedure, the circumflex collaterals were preserved, and the deep dorsal venous valves were not disrupted by a stripper. After the inferior epigastric artery was brought to the penile root through the subcutaneous tunnel, an end-to-side anastomosis was performed with the proximal part of the deep dorsal vein. 7–0 polypropylene suture were used according to a standard microsurgical procedure. After the anastomosis, the deep dorsal vein was ligated proximal to the arteriovenous anastomosis. The procedure was performed under optical magnification (×2.5) to prevent neurovascular bundle damage. In the postoperative period, intravenous heparin (5000 IU/day) was prescribed for 3 days, 75 mg/day dipyridamole and 300 mg/day acetylsalicylic acid daily for three months. He was warned not to have sexual intercourse for 2 months after the operation. Face-to-face interviews were conducted with all the patients at the postoperative third month. During these follow up, the patients were re-evaluated with the IIEF-5 and 15 questionnaire, and CTA scanning was performed.

### CTA protocol

**Fig. 1:**
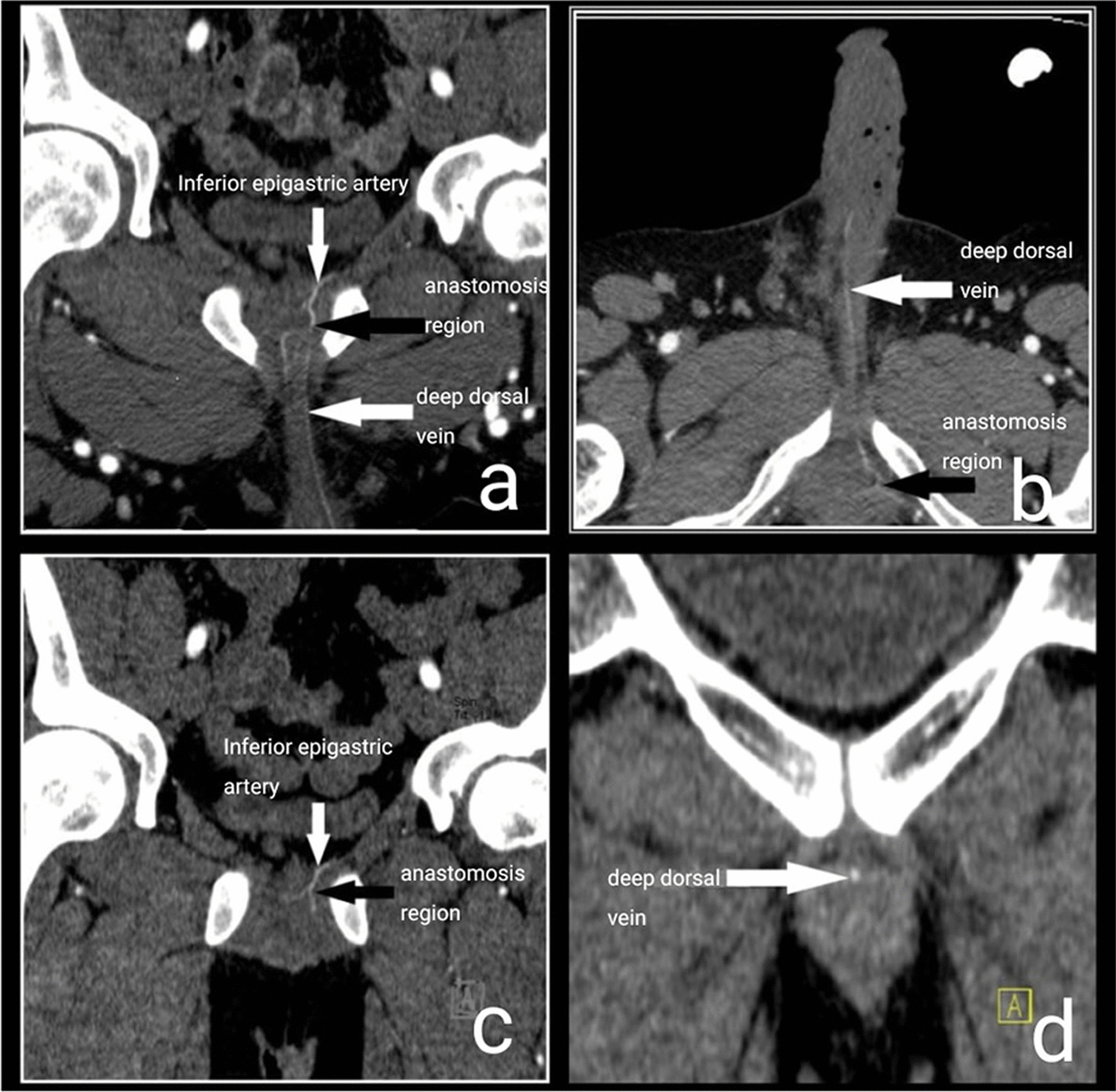
**a**–**d** Postoperative pelvic CT angiography images a 30-years-old male. **a** The image of the left inferior epigastric artery, the anastomotic region, and the arterialized deep dorsal vein and its branch in the coronal cutaway of the penis, **b** The image of the anastomosis region and deep dorsal vein in the transverse cutaway of the penis, **c** The image of left inferior epigastric artery and anastomosis region in the coronal cutaway of the penis, **d** The image of arterialized deep dorsal vein. It is observed that the deep dorsal vein was contrasted in the coronal cutaway of the penis

**Fig. 2:**
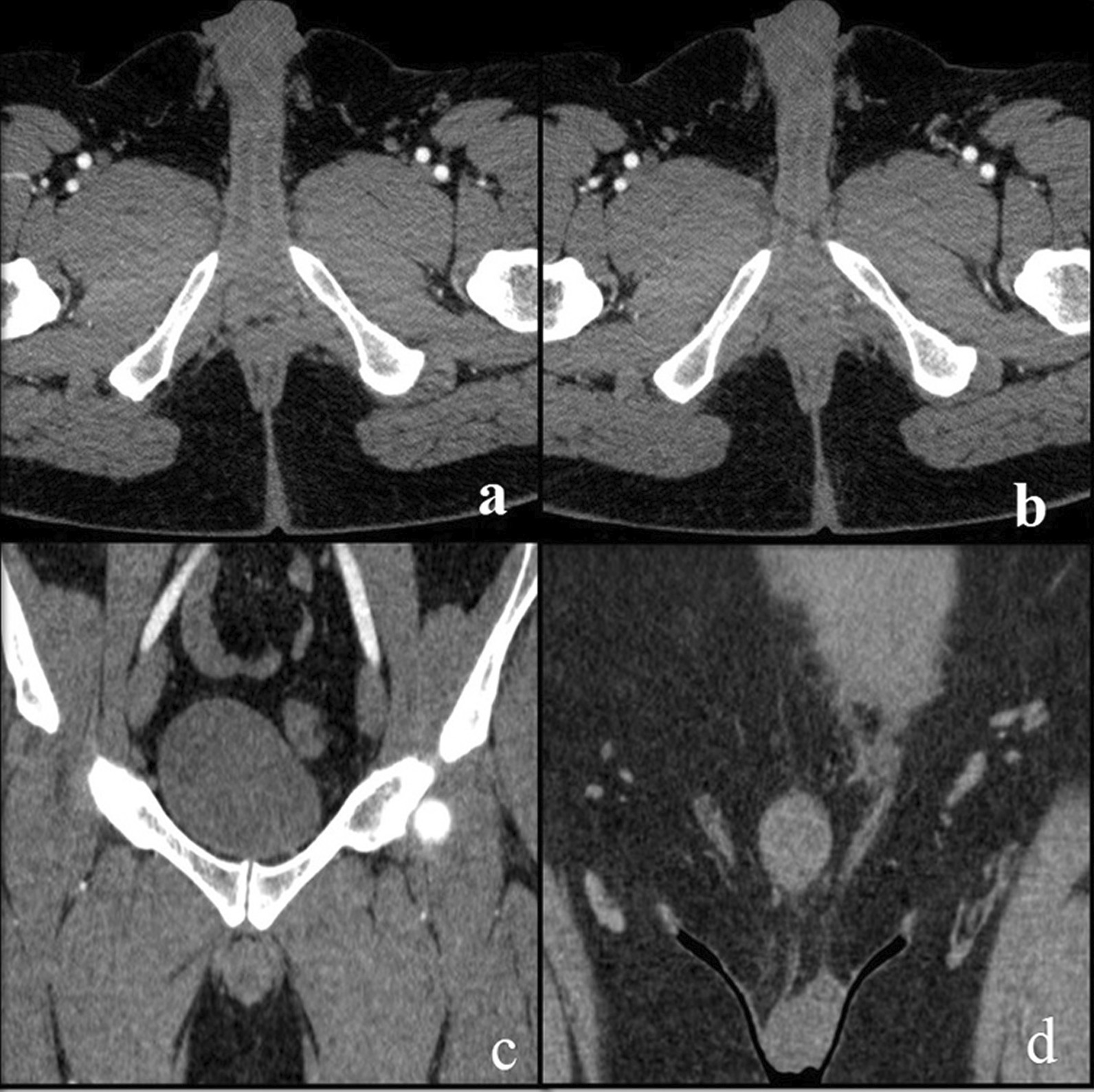
**a**–**d** Postoperative pelvic CT angiography images a 42-years-old male. Penil vascularity was prominently diminished and vascular structures were not observed

CTA was performed in all the patients at the postoperative third months. Cases with normal creatinine levels and no contrast allergy were included in the CTA planning. To improve image quality, patients were fasted for eight hours before the procedure. Papaverine hydrochloride at a dose of 60 mg was administered intra-cavernosally 10 min before CTA scanning. In this way, it was aimed to better evaluate the vascular structures by creating a penile erection. Before CTA scanning, a vascular access was made in the patient's forearm, preferably through the basilic vein or the cephalic vein, with a 22 gauge branule. Then, the patient was placed in a supine position centered on the CTA scanning stretcher and with the hands up. The image area to be examined in the CTA scanning has been determined. This area was determined as the distance between the umbilicus and the distal of the penis. Then, using an automatic injector pump, iodinated contrast material (Iopromide, Ultravist®, Schering, Germany) was given to the patient intravenously at a dose of 2 mg/kg and at a flow rate of 3 ml/s. Then, arterial phase pelvic CT angiography with 2 mm slice thickness was performed with a 64-detector, multi detector CT machine (Aquilion 64, Toshiba®, Tokyo, Japan). After CT angiography examination, sagittal and coronal reformatted images (slice thickness: 1 mm) were obtained. CT images were evaluated by an experienced radiologist (Figs. 1a–d and 2a–d].

#### Analysis of outcomes and statistics

All the patients completed the IIEF-15 questionnaire preoperatively and during the postoperative follow-up. In addition, IIEF-5 and EF domain scores were recorded preoperatively and postoperatively. All the statistical analyses were performed using SPSS software for Windows (version 21.0, SPSS Inc, Chicago, Illinois, USA). Continuous data were expressed as mean ± standard deviation (SD). Student’s t-test was used for the comparison of the averages, and the chi-square Fischer test was undertaken to compare the categorical variables. Categorical data were expressed as values and percentages. *P* < 0.05 was considered as statistically significant. In the power analysis undertaken, the dependent-samples t-test was used to examine the preoperative and postoperative differences. The smallest effect size was calculated as 1.37. Using G*Power software package v. 3.1, the power of the study was determined as 95.5% with an effect size of 1.37, significance level of 0.05, and sample size of 22 patients.

## Results

Penile revascularization surgery was performed on 31 patients in Group 1, 28 in Group 2, and 19 in Group 3. These cases were evaluated using PCDU, CC-EMG, and cavernosometry and classified according to the etiology of ED. There were 42 cases of arterial insufficiency, 17 cases of venous insufficiency, and 19 cases of both arterial and venous insufficiency. In preoperative PCDU, the mean PSV values of the right and left cavernosal arteries, were determined as 13.41 and 14.16 cm/s, respectively for the patients with arterial insufficiency, 38.42 and 41.20 cm/s, respectively for with venous insufficiency, and 16.33 and 15.84 cm/s, respectively for those with both arterial and venous insufficiency. In preoperative PCDU, the mean EDV values of the right and left cavernosal arteries, were found to be 3.41 and 2.16 cm/s, respectively for the patients with arterial insufficiency group 8.50 and 9.35 cm/s, respectively for those with venous insufficiency, and 7.65 and 8.33 cm/s, respectively for those with both arterial and venous insufficiency. Veno-occlusive dysfunction was detected in 17 cases in cavernosometry performed after the complete compression of the cavernosal smooth muscles following the intra-cavernosal papaverine injection in CC-EMG. Based on the preoperative IIEF-5 scores, two patients had mild ED, six had mild to moderate ED, 22 had moderate ED, and 48 had severe ED (Table 1).

**Table 1:** Classification of the degree of ED according to the patients’ preoperative IIEF-5 scores

	IIEF-5 scores	20–40 years	41–60 years	> 60 years
No ED	(22–25)	0	0	0
Mild ED	(17–21)	0	2 (2.56%)	0
Mild & Moderate ED	(12–16)	2 (2.56%)	4 (5.12%)	0
Moderate ED	(8–11)	7 (8.97%)	10 (12.8%)	5 (6.41%)
Severe ED	(5–7)	11 (14.1%)	20 (25.6%)	17 (21.79%)

IIEF: International index of erectile function, ED: erectile dysfunction

For the patients whose anastomotic region and deep dorsal veins were visualized on CTA (Fig. 1a–d), the IIEF-5 and IIEF-15 scores were calculated as 8.66 and 19.77, respectively in Group 1, 9.46 and 21.15, respectively in Group 2, and 4.0 and 12.66, respectively in Group 3 in the preoperative period and 15.66 and 38.33, respectively in group 1, 17.23 and 39.46, respectively in group 2 and 5.40 and 17.33, respectively in Groups 3 in the postoperative period. The rate of increase in the IIEF-5 score was 80.8% in Group 1, 82.1% in Group 2, and 35.0% in Group 3. Concerning the IIEF-15 scores, there was an increase of 93.8% for Group 1, 86.5% for Group 2, and 36.8% for Group 3.

In cases where anastomosis area cannot be observed in CTA (Fig. 2a–d), the IIEF-5 and IIEF-15 scores were 6.0 and 21.0, respectively in Group 1, 9.50 and 22.25, respectively in Group 2, and 4.80 and 11.60, respectively in Group 3 in the preoperative period, and 7.40 and 26.0, respectively in Group 1, 12.0 and 27.75, respectively in Group 2, and 5.40 and 12.80, respectively in Group 3 in the postoperative period. The rate of increase in the IIEF-5 scores was 23.3% in Group 1, 26.3% in Group 2, and 12.5% in Group 3. The IIEF-15 score increased by 23.8% for Group 1, 24.7% for Group 2, and 10.3% for Group 3 (Table 2). When the preoperative and postoperative IIEF-5 scores were compared, it was found that 39 of the 51 patients with severe or moderate ED in cases where anastomosis area can be observed had mild-moderate or mild progression or no ED. In cases where anastomosis area cannot be observed, only one of the 19 cases with severe and moderate ED had mild to moderate ED, while no improvement was found in the remaining 18 patients.

**Table 2:** Changes in the preoperative and postoperative IIEF scores of the patients with and without the visualization of the anastomotic opening CTA (IIEF: International Index of Erectile Function)

	Anostomosis open (n = 56)			Anostomosis not open (n = 22)			*P* value*
	Preop. IIEF 5/15	Postop. IIEF5/15	IIEF 5/15 rate of increase	Preop. IIEF 5/15	Postop. IIEF 5/15	IIEF 5/15 rate of increase	
Group 1 (20–39 year)	8.66/19.77	15.66/38.33	80.8%/93.8%	6.0/21.0	7.4/26.0	23.3%/23.8%	< 0.05
Group 2 (40–59 year)	9.46/21.15	17.23/39.46	82.1%/86.5%	9.5/22.25	12.0/27.75	26.3%/24.7%	< 0.05
Group 3 (> 60 year)	4.0/12.66	5.4/17.33	35.0%/36.8%	4.8/11.60	5.4/12.80	12.5%/10.3%	< 0.05

*Statistical significance

The preoperative total IIEF-EF domain scores were calculated as 6.4, 4.8 and 5.9 in Groups 1, 2 and 3, respectively. For the patients with anastomotic patency in the CTA performed in the postoperative follow-up, the total IIEF-EF domain scores were 18.16 in Group 1, 21.13 in Group 2, and 8.65 in Group 3 while these values were 9.35 in Group 1, 13.6 in Group 2, and 6.62 in Group 3, in cases where anastomosis patency cannot be observed. According to the postoperative IIEF-5 results, it was observed that there was at least one degree improvement in the severity of ED in a total of 47 cases (60.25%). While 92% of the patients in group 1 with open anastomosis had at least one degree of improvement in the severity of ED, this rate was found to be 25% in patients in group 3. These rates were found to be much lower in cases whose anastomosis was not open (Table 3). Although the anastomos is patency was observed in eight cases, a sufficient IIEF increase was not observed. However, a penile prosthesis implantation decision was not rushed. During the follow up of these patients four were observed to have gradually increased IIEF values over the postoperative six to 12 months. In the remaining four cases, the expected benefit from penile revascularization was not observed and penile prosthesis implantation was recommended to these cases.

**Table 3:** Classification of cases with at least one degree improvement in ED severity according to age groups and anastomotic patency

	Anastomosis open (n = 56)	Anastomosis not open (n = 22)
Group 1 (n = 31)	23 Subjects (+)	1 Subjects (+)
	2 Subjects (−)	5 Subjects (−)
	RI: 92.0%	RI: 16.6%
Group 2 (n = 28)	19 Subjects (+)	1 Subjects (+)
	4 Subjects (−)	4 Subjects (−)
	RI: 82.60%	RI: 20.0%
Group 3 (n = 19)	2 Subjects (+)	1 Subjects (+)
	6 Subjects (−)	10 Subjects (−)
	RI: 25.0%	RI: 9.09%

(n): Number of subjects, (+): at least one degree improvement in ED severity, (−): no improvement in ED severity, (RI): rate of Improvement

## Discussion

In cases where oral pharmacotherapy and intracavernosal injections are not effective in ED treatment, penile revascularization can be performed as a third-line treatment [11]. Surgical modifications can be summarized as modifications of anastomoses between the IEA and the dorsal penile artery or the deep dorsal vein, direct anastomosis to cavernous bodies, or end-to-end (triple-stapled) anastomosis between the IEA, the dorsal artery and the vein [12–14]. Recently, laparoscopic and robot-assisted revascularization techniques have been also applied, but in these studies, the number of patients was low and surgical experience was not sufficient to generalize the outcome to a wider population [15, 16].

The aim of penile revascularization surgery is to raise the amount of blood to the cavernous bodies to increase oxygenation, maintain smooth muscle structure and achieve spontaneous physiological erection. The first study on penile revascularization was published in 1972 by Michal et al. [17]. Later, Goldstein reported an 80% success rate with the same method in younger patients with ED secondary to pelvic trauma accompanied by the localized obstruction of the internal pudendal or penile arteries [18]. Virag et al. [19] performed an aortic anastomosis between the IEA and the deep dorsal vein and reported normal erection in 49% of the patients and improved erection in 20%. The results of long-term follow-up studies performed over the last three decades using different techniques indicate success rates varying from 25 to 80% [20–28]. In a study of a large case series with a long follow-up period, Kayigil et al. reported high success rates for penile revascularization in patients with no risk factors [29].

In the current study, an end-to-side anastomosis was performed between the IEA and the deep dorsal vein of the penis. This directed the arterial flow in the deep dorsal vein into the cavernous bodies through emissary veins and aimed to improve EF by increasing tissue oxygenation. Penile revascularization surgery does not produce the same result in all patients with ED caused by vascular insufficiency. For successful outcomes, patency of the anastomosis established to increase the blood flow to the cavernous tissue should be maintained in the postoperative period. In a study that used selective pudendal angiography to assess the anastomotic patency in cases that had undergone penile revascularization surgery, no association was reported between anastomotic specificity and subjective patient satisfaction [30]. However, in that study, arterio-arterial anastomosis was used and the selected imaging technique was highly invasive and had a high complication rate. To evaluate patients with ED prior to the revascularization procedure magnetic resonance angiography (MRA) and penile angiography were compared and the latter was reported to be superior. However, this method is invasive and expensive, and has the disadvantages of requiring follow-up after surgery. In the same study, the proximal iliac and pudendal arteries were reliably shown with MRA but the visualization of the distal pudendal and penile arteries was limited. This was attributed to artifacts due to bowel movements and the limited spectral resolution [31].

In the present study, CTA was performed at the postoperative third month to determine the anastomotic patency. Offering excellent anatomical detail by imaging at all angles, this technique allows for the easy identification of patients that require endovascular treatment. CTA is a fast and non-invasive imaging method for defining vascular diseases and demonstrating the vascular anatomy and the relationship between non-vascular diseases and vascular structures. Contrary to conventional angiography, CTA can be performed with the peripheral intravenous injection of contrast agents (generally into the basilic or cephalic vein) and has been defined as a first-choice imaging technique for the diagnosis of vascular diseases. This technique can also be used to follow up anastomotic patency in previously diagnosed cases as described in this article. The brevity of the imaging period and patient follow-up after the procedure lasting only minutes are important advantages of CTA over MRA, particularly for patients with claustrophobia. Considering the long duration of MRA and the possibility of artifacts in images due to the patient’s movements, CTA presents as a more favorable method. We consider that the detailed imaging of vascular and other anatomical structures with CTA can be attributed to the vasodilating effect of papaverine administered before the procedure.

CTA has attracted the attention of researchers in the diagnosis of conditions such as developmental vascular anomalies and atherosclerosis [32–34]. The characteristic CTA findings are of great importance in evaluating the severity of disease and patient response to treatment [35]. In a study on the use of CTA in patients with ED, the internal pudendal artery, a branch of the internal iliac artery providing penile erection, was assessed in terms of its anatomical variations and effect on the age of developing ED. According to the results, variations of the internal pudendal artery were seen in about 50% of patients and were considered to play a role as a congenital factor, especially in the early etiology of ED. It was also reported that ED might develop approximately 10 years earlier in patients with bilateral anatomic variations compared to those with a normal bilateral anatomy [36]. Beijer et al. [37] suggested that angiography should be considered as the gold standard in the detailed imaging of arterial diseases, and CTA can be used as a first-line method in patients with arterial disease. In a study in which 73 patients were evaluated, CTA was stated as a promising and effective method in the diagnosis of ED of vascular origin [6]. In another study, it was emphasized that CTA was a reliable method in the diagnosis of venous ED, showing the exact location of venous leakage in clinical examination, through clearer images, with further advantages of lower contrast agent and radiation doses, and faster examination compared to X-ray penile angiography [8]. In the literature, there is no study reporting CTA being performed to demonstrate vascular structures and anastomotic patency after penile revascularization. We consider that the current study provides a better understanding of the efficacy of penile revascularization surgery in the treatment of ED and contributes to this surgical technique. In our study, the IEA, deep dorsal vein and anastomotic region were better evaluated using CTA with the papaverine injection in the postoperative period. The statistical analysis revealed that in patients with the visualization of the deep dorsal vein and anastomosis patency, the increase in the IIEF scores and patient satisfaction were significantly higher. In the elderly patient group, the success rates were found to be lower. It is difficult to recommend penile revascularization surgery as a standard approach in older patients. However, it can be offered as an alternative in selected cases who have not benefited from medical treatments and are not willing to implant a penile prosthesis. In these cases, the patient should be told that the chance of success is low. In our study, the patients with an insufficient increase in the IIEF scores and no visualization of the anastomosis patency were recommended penile prosthesis implantation. However, in cases with a visible anastomotic patency in CTA despite an insufficient increase in IIEF scores, prosthetic implantation was not immediately recommended and it was considered more appropriate to continue the treatment with phosphodiesterase 5 inhibitors for a certain period.

As in all other techniques, the most reliable way of performing a CTA evaluation with the correct indications is to understand the positive and negative aspects of the method and its technical capacity, and applying it in cases where it will provide additional information that will influence the diagnosis and treatment protocol of the patient. Despite offering more anatomical details, CTA has certain disadvantages due to the use of contrast materials and exposure to radiation. However, with the advances in technology, CTA devices now have a higher scanning capacity and require a shorter time for imaging; thus, the duration of exposure to radiation has been minimized.

## Conclusion

In ED cases, penile revascularization surgery can be performed before invasive procedures such as penile prosthesis implantation. The scoring systems used to demonstrate the effectiveness of this surgery in the postoperative period are mostly subjective. Therefore, in this study the similarity of the findings obtained from contrast enhanced CTA with the IIEF scores suggest that CTA is an objective method to identify the success of the surgery and follow-up patients in the postoperative period. It was also observed that CTA could be used as a pre-evaluation before implantation in prosthesis candidates who do not benefit from other treatment options, especially at younger ages. We consider that further studies with a larger case series will shed more light on the effectiveness and advantages of CTA.

## Data Availability

The datasets used and/or analysed during the current study are available from the corresponding author on reasonable request.

## Abbreviations

CTA: Computerized tomography angiography
ED: Erectile dysfunction
IIEF: International index of erectile dysfunction
EF: Erectile function
CC-EMG: Corpus Cavernosum electromyography
PCDU: Penile color Doppler ultrasonography
PSV: Peak systolic velocity
EDV: End diastolic velocity
IEA: Inferior epigastric artery
CEA: Cavernosal electrical activity
MRA: Magnetic resonance angiography
